# One year after the approval of Lecanemab in Japan

**DOI:** 10.1002/alz70859_100084

**Published:** 2025-12-25

**Authors:** Yuki Kujuro

**Affiliations:** ^1^ Tachikawa Hospital, Tachikawa, Tokyo Japan

## Abstract

**Background:**

Since the approval of Lecanemab in Japan on September 25, 2023, for mild cognitive impairment (MCI) and mild dementia due to Alzheimer's disease (AD), the number of dementia‐related phone inquiries at our hospital has increased by 1.7 times over a period of 6 months (from 457 inquiries before to 759 inquiries after). Due to the public health insurance system and the high‐cost medical expense system in Japan, patients have equal access to Lecanemab treatment. We will present how we have prepared our outpatient services, our experience with using the drug, and the arrangements for continued administration after 6 months.

**Method:**

We conducted a retrospective data analysis of 58 patients who were treated with Lecanemab between January and December 2024 at our Center for Dementia‐Related Diseases in Tokyo. All patients have been confirmed to be amyloid pathology positive by either cerebrospinal fluid (CSF) or amyloid PET.

**Result:**

82% of the patients who started Lecanemab were referred by general practitioners, and 40% of those referrals specifically requested consideration of Lecanemab treatment. After scheduling an outpatient appointment, a head MRI and neuropsychological tests were performed at our facility before the first medical examination. Clinical characteristics of patients treated with Lecanemab were as follows: age at 1st Lecanemab infusion 76.2±8.5 years (54‐92 years), 69% female, 96.6% Japanese (1.7% Chinese, 1.7% Korean), MMSE 24.6±2.0 (22‐30), CDR‐J 0.5: 92%, and 1: 8%, CSF Aβ42/Aβ40 ratio 0.049±0.009, Amyloid PET Centiloid scale 75.2±21.1. None of the patients had amyloid‐related imaging abnormalities (ARIA) ‐E, 6.9% had ARIA‐H (5.2% asymptomatic mild, 1.7% asymptomatic moderate), 1.7% had mild infusion reaction. Following the initial 6 months of Lecanemab administration, continuous infusions can be carried out at facilities that meet certain criteria. Consequently, we have referred 11 patients to other facilities.

**Conclusion:**

Consistent with prior evidence, Asian patients tend to have less ARIA side effects. Due to the high demand of Lecanemab treatment, it is necessary to promptly refer patients to continuous administration facilities after the initial 6 months of administration.

